# Risk factors for incomplete excision of cutaneous squamous cell carcinoma: A single-center study performed on 1082 excisions in Northern Italy

**DOI:** 10.1016/j.jpra.2025.02.007

**Published:** 2025-03-06

**Authors:** S.C. van Helmond, S. Cozzi, B. Breugelmans, D. Codazzi, L. Valdatta, M. Carminati

**Affiliations:** 1 Plastic and Reconstructive Surgery Unit, ASST Papa Giovanni XXIII, Bergamo, Italy; 2 Faculty of Health, Medicine and Life Sciences, Maastricht University, Maastricht, the Netherlands; 3 Department of Biotechnology and Life Sciences, University of Insubria, Varese, Italy

**Keywords:** keratinocyte carcinomas/ non-melanoma skin cancer, cutaneous squamous cell carcinoma, excision margin, margin status, incomplete excision, risk factor

## Abstract

Cutaneous squamous cell carcinoma, which makes up for 25% of keratinocyte carcinomas, is the second most frequent skin cancer worldwide. Surgical excision via a clinical and microscopic complete resection is the treatment of choice. Incomplete excisions carry the risk of local recurrence, deep subclinical progression, and metastasis. This retrospective cohort study aimed to investigate the risk factors associated with the incomplete excision of cutaneous squamous cell carcinoma. This series included 837 patients who underwent surgical treatment for 1082 primary cutaneous squamous cell carcinoma in the Plastic Surgery Department of the Papa Giovanni XXIII Hospital in Bergamo, Northern Italy between 2012 and 2021. Patient-, procedure-, and tumor characteristics were collected and analyzed. Incomplete excision rate was 11.8% (n=128). The Pearson chi-squared test and univariable logistic regression showed tumor diameter [cm] (p<0.001), tumor thickness [mm] (p<0.001), tumor location (ear p=0.006, peri-orbital, p=0.029), differentiation grade (G3=0.005), infiltration level (hypodermis, p<0.001; muscle, p=0.013; bone/ cartilage p<0.001), presence of perineural invasion (p=0.041), ulceration (p=0.010), no prior diagnostic biopsy (p=0.041), and additional samples taken (p<0.001) with additional samples not free of tumor cells (p<0.001) to be significant risk factors/ predictors for incomplete excision. Risk factors should be considered in the management of cutaneous squamous cell carcinoma. This study documented several key contributions and confirmations regarding the risk factors associated with incomplete excision in cutaneous squamous cell carcinoma by comprehensively analyzing of one of the largest cohort studies in the field.

## Introduction

Skin cancer is one of the three most common cancer types in Italy.^1^ There is a global increasing trend in skin cancer prevalence over the recent years.^2^, ^3^, ^4^ The World Health Organization declared that this increase involves melanoma skin cancer and keratinocyte carcinomas.^5^ Cutaneous squamous cell carcinoma (cSCC), which makes up for 25% of the keratinocyte carcinomas, is the second most frequent skin cancer worldwide.^6^^,^^7^ The first line treatment for cSCC is surgical excision with a clinical and microscopic complete resection. A surgical margin contains clinically normal-appearing tissue around the tumor. For low and high risk cSCC, different surgical margins are recommended.^8^ Incomplete excisions may cause local recurrence, deep subclinical progression, and metastasis.^9^ A re-excision surgery is recommended for incomplete excisions and also in the event of close margins.^10^ Several studies investigated the risk factors associated with incomplete margin status in cSCC excision. To this date, published research on margin status shows high heterogeneity in study designs. Additionally, only few studies involved large populations with cSCCs (>1000). This results in vast variations in incomplete excision rates, risk factors weight, and scarce conclusiveness.^11^

In the present retrospective study, the authors collected and analyzed a substantial amount of data on tumor-, procedural- and patient-characteristics. To the authors’ knowledge, this study is one of the largest cohort studies on incomplete excision of cSCC and includes the largest study population within the specific field of plastic surgery.^12^, ^13^, ^14^ This study aimed to identify the prognostic factors for incomplete excision. From a clinical perspective, the endpoint is to improve cSCC treatment in daily practice. By considering such risk factors prior to primary surgery, incomplete excision and re-excision surgery can be minimized.

## Patients and methods

### Ethics

Ethical approval: Not required.

### Study design

This retrospective study included all histopathologically-verified primary cSCCs that were surgically treated using wide local excision (WLE) between January 1, 2012 and December 31, 2021. All surgical excisions were performed by the plastic surgery unit of the ASST Papa Giovanni (PG) XXIII Hospital in Bergamo, Northern Italy. Surgical margins were based on the Italian Medical Oncology Association (AIOM) guidelines with cSCC risk categories defined by American (NCNN) guidelines applicable to the year of surgery (AIOM margin guidelines: low risk ≥4 mm, high risk ≥6 mm). The specimen were examined and reported on by the Department of Pathology in the PGXXIII, at the same center. All histologically verified cutaneous invasive, in situ, and keratoacanthoma-like cSCCs were included consecutively. Procedures that were solely intended to remove the tumor completely were considered; however, re-excisions and palliative excisions were excluded. Recurrent cSCCs and cSCCs suspected for local recurrence were excluded. Histopathological reports were retrieved from a database provided by the local epidemiology department, these were used to compile the clinicopathological data (supplementary table 1). The American (NCNN) guidelines of the year of cSCC surgery were used for defining the histopathological variables.

Tumor locations with widely varying recording options were simplified into groups and subgroups. (supplementary figure 1) A facial subdivision was made to regroup tumor sites into high-risk zones. (supplementary figure 2). Margin status was classified as complete (i.e., free, clear margins) or incomplete (i.e., not free, positive margins), in case of a second lesion present at the margin site (different from the primary cSCC) the distance from tumor and specimen margins was classified as missing. When a diagnostic biopsy is performed, the clinical indication is left blank.

### Statistical analysis

Analyses were conducted using IBM SPSS Statistics version 27. Data were summarized using descriptive analysis. Not-normal distributed continuous variables are expressed as the median with range. The Pearson chi-squared test was used to assess the association between surgical margin status (complete/incomplete) and categorical variables. For the association between surgical margin status and continuous variables, the Mann–Whitney U test was used. Univariable logistic regression analyses were performed using dependent variable margin status [0=incomplete, 1=complete]. The Box-Tidwell test for linearity was performed before analyzing continuous variables in univariable logistic regression. Missing data were reported and included.

## Results

**Figure 1:**
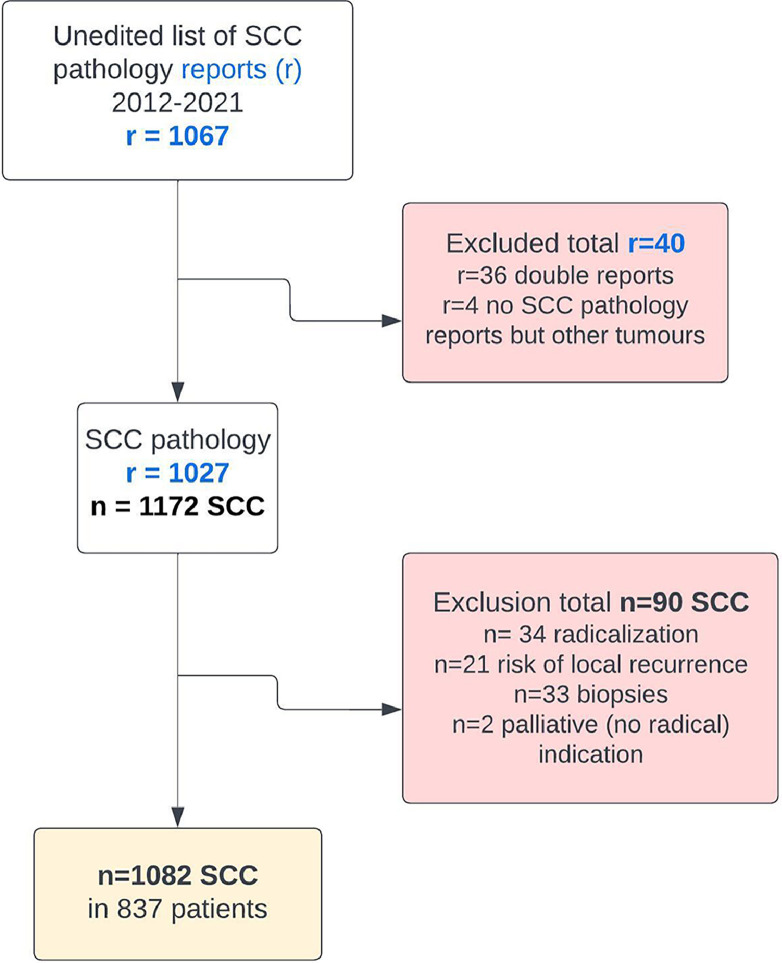
Flowchart of study

**Figure 2:**
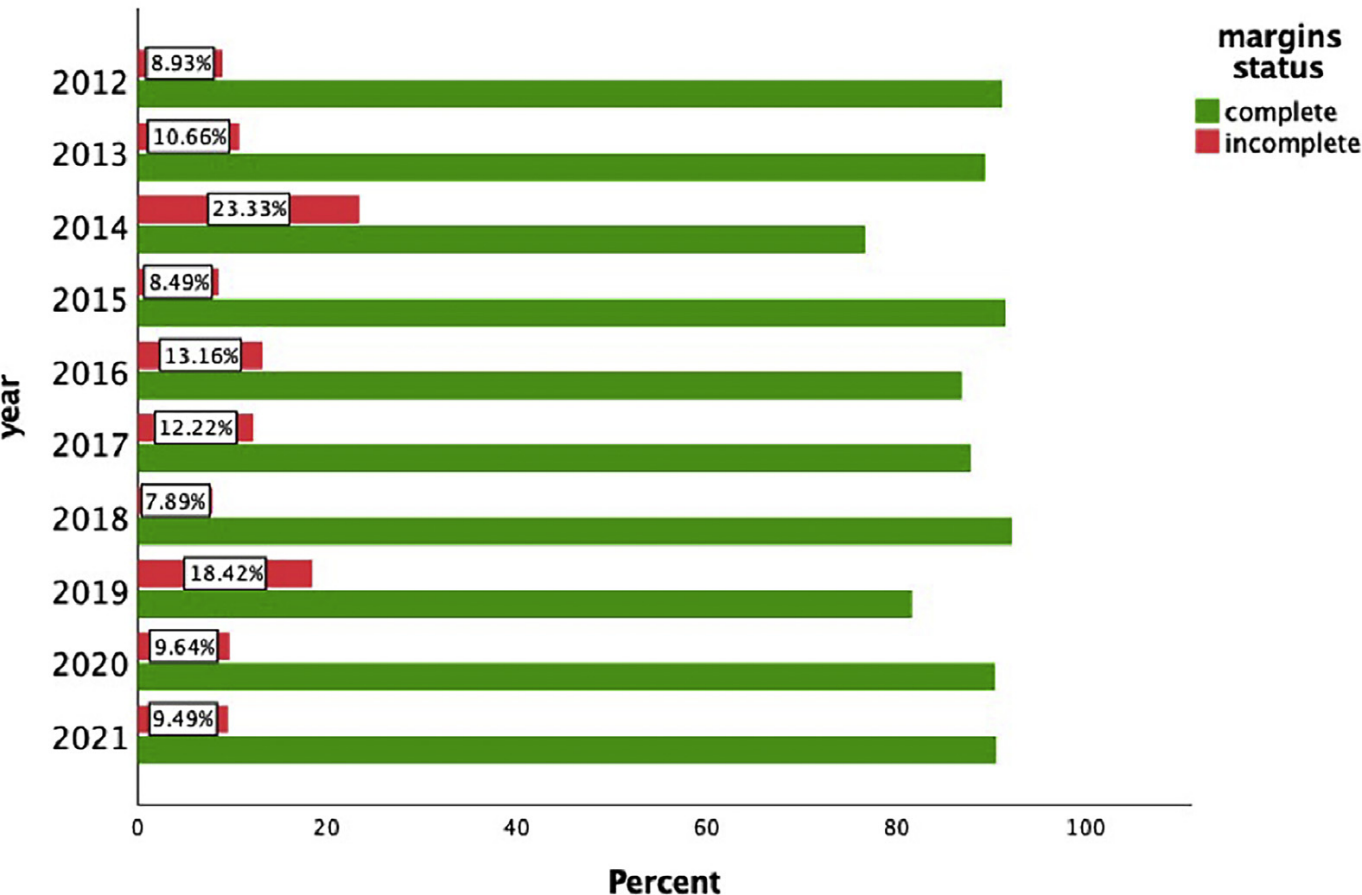
Incomplete excision rate for year of primary surgery

**Figure 3:**
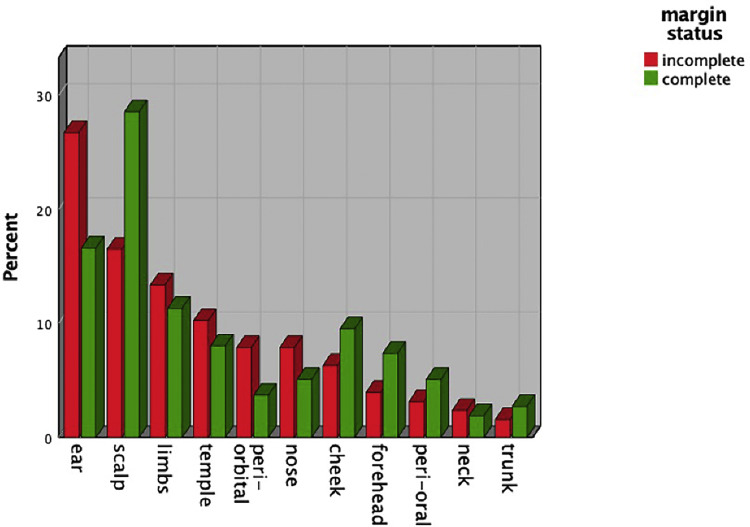
Distribution in percentages of subdivided tumour sites for excision type

**Figure 4:**
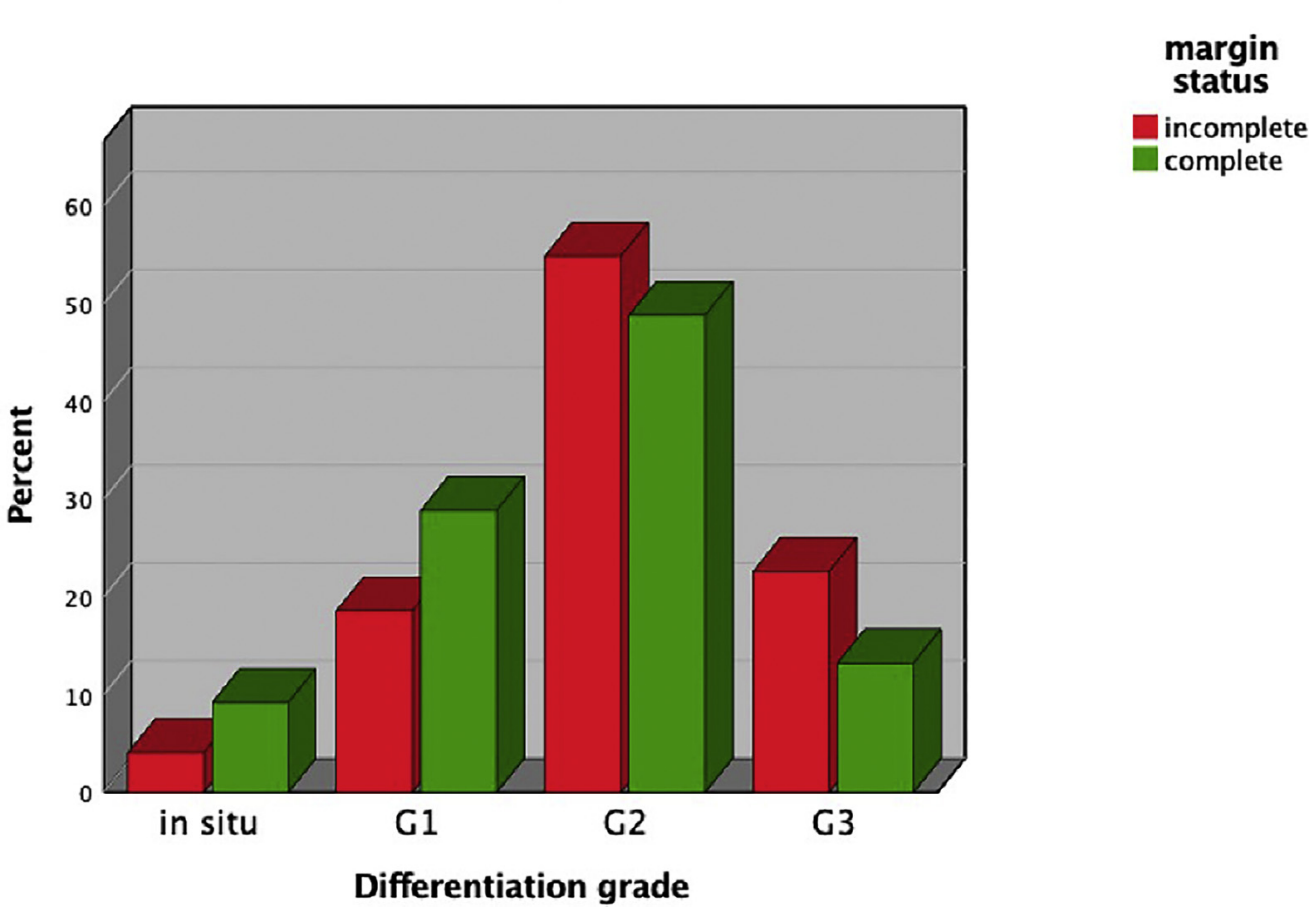
Distribution in percentages of differentiation grade for excision type

**Figure 5:**
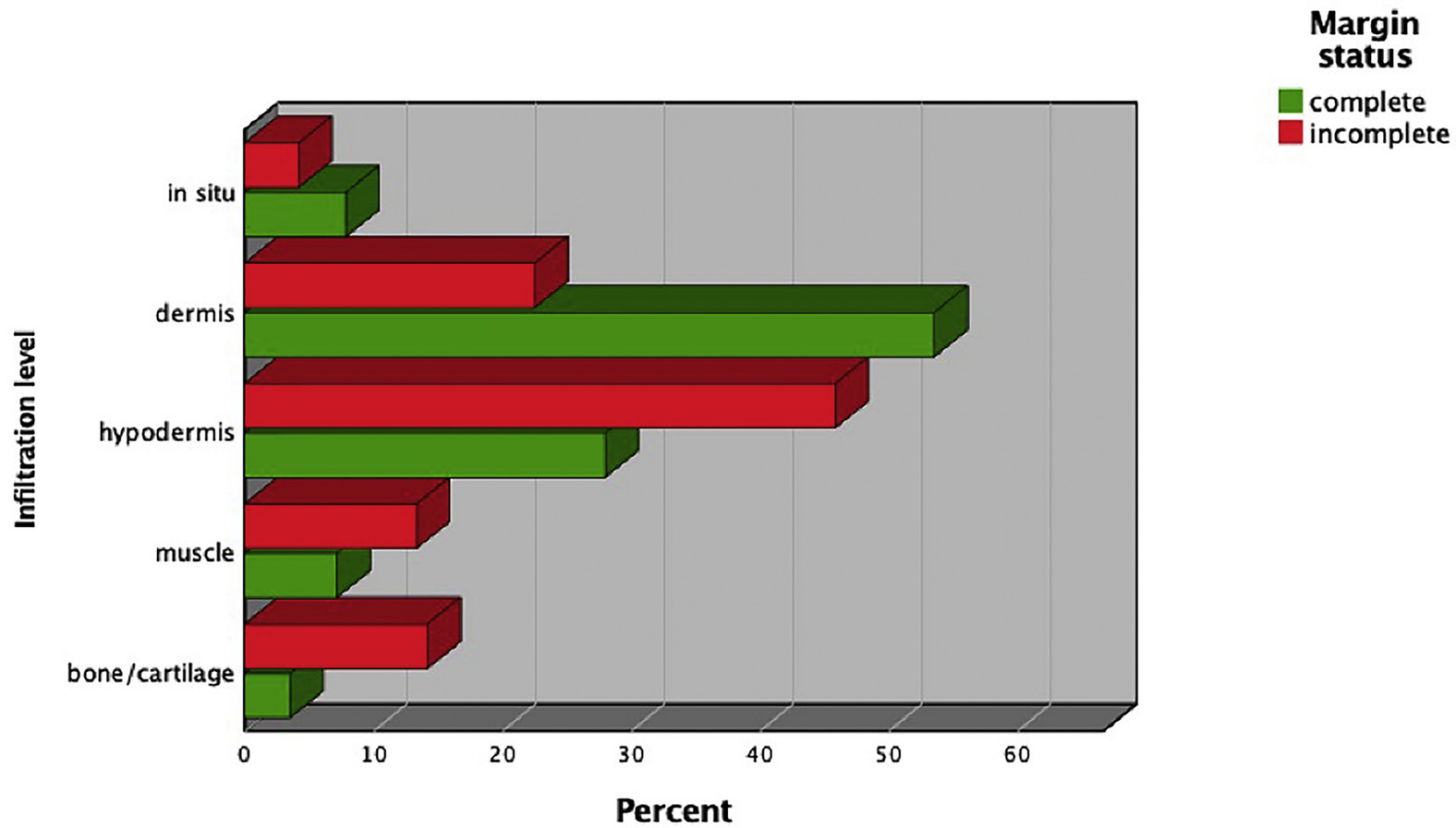
Distribution in percentages of infiltration level for excision type

**Figure 6:**
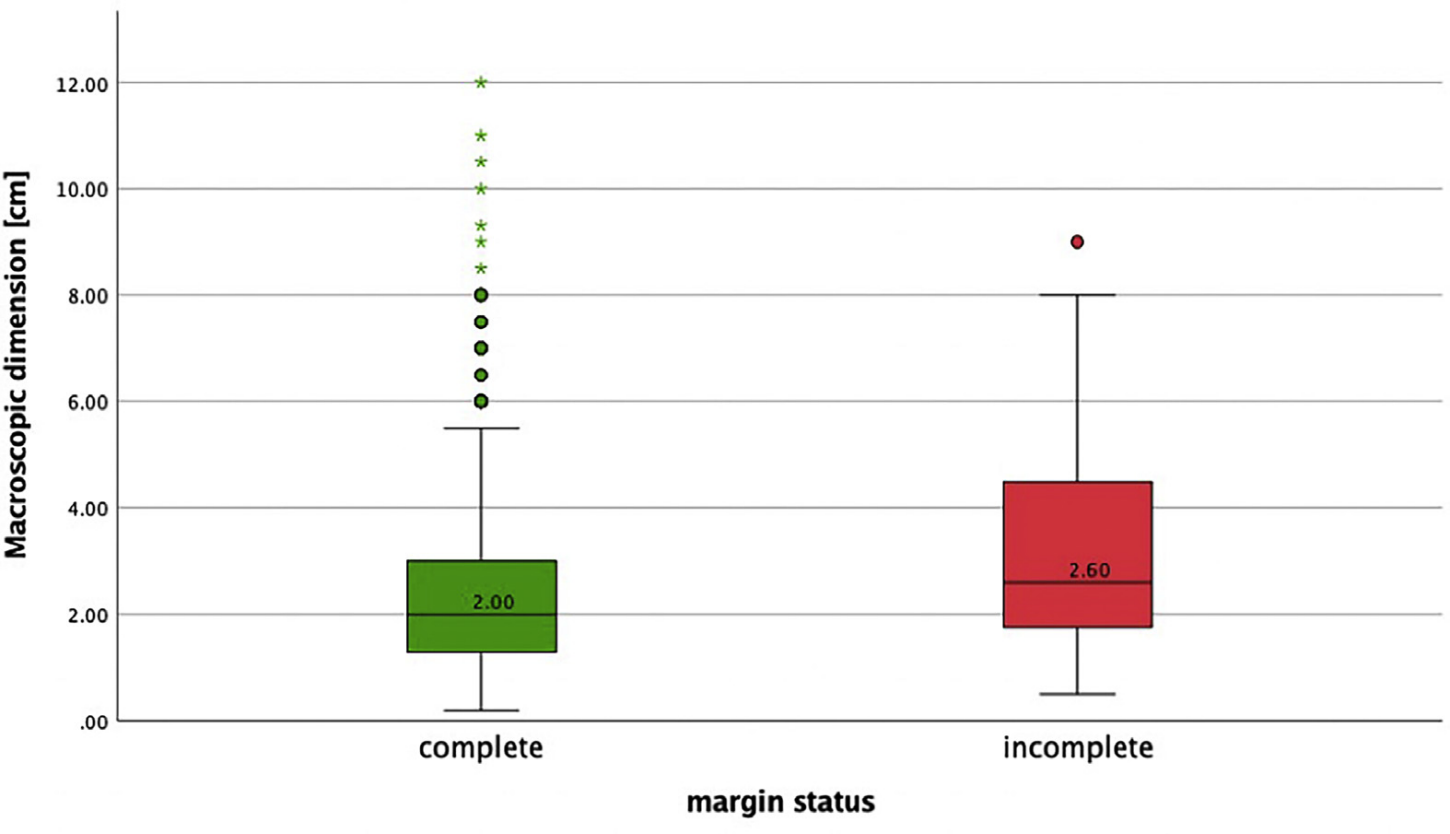
Boxplot distribution of macroscopic dimension (cm) for margin status

**Figure 7:**
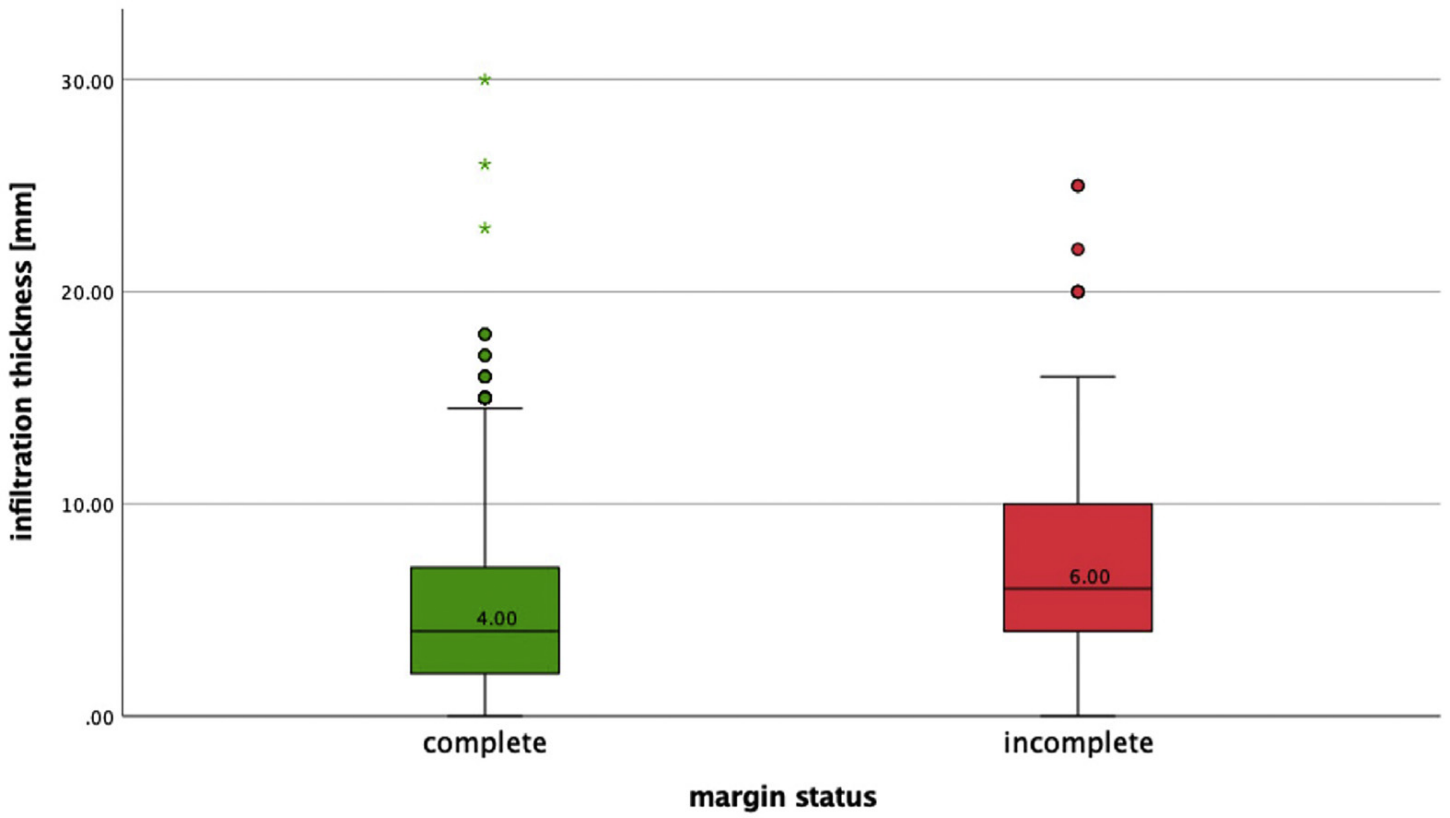
Boxplot distribution of infiltration thickness (mm) for margin status

In total, 1082 cSCCs in 837 patients were included (Figure 1). This series included 214 women (25.6%) and 623 men (74.4%). The median age of our study population was 81 years (range 36-99 years). Median age by gender was 85 years (range 41-99 years) for women and 79 years (range 36-98 years) for men. The overall incomplete excision rate was 11.8% (n=128) with 87.4% (n=946) completely excised and 0.7% (n=8) missing. Incomplete excision rates are separated for the year of primary surgery (Figure 2). Among the incomplete excision specimen, cancer cells were present at the deep-, lateral- or both margins in 46.6%, 41.4%, and 12.0% of the cases, respectively. The lesions were most frequently located in the head and neck (H&N) area (85.7%), followed by limbs (11.6%) and trunk (2.6%) with the scalp (27.1%), ear (18.0%), and limbs (11.6%) being the three most frequent subgroups (Table 1, Figure 3). The G2 differentiation grade (47.8%) and infiltration level at the dermis (45.2%) were the most frequent (Table 1). Distributions [%] of differentiation grade, infiltration level, and tumor site subgroups were divided for margin status (Figures 3–5). Missing data were analyzed (Table 2), Figure 4. The median tumor diameter was 2.00 cm (range 0.20-13.00 cm) (Figure 6). The median tumor thickness was 4.00 mm (range 0.00-38.00 mm) (Figure 7).

**Table 1:** Tumor characteristics for cSCCs excised at the Plastic Surgery Department of the Papa Giovanni XXIII Hospital between 2012-2021.

Tumor characteristics	Frequency	Percentage (%)
*Tumor site subgrouped*		
Cheek	98	9.1
Ear	195	18.0
Forehead	75	6.9
Limbs	125	11.6
Neck	21	1.9
Nose	58	5.4
Peri-oral	52	4.8
Peri-orbital	45	4.2
Scalp	293	27.1
Temple	90	8.3
Trunk	28	2.6
*Differentiation grade*		
In situ	90	8.3
G1	289	26.7
G2	517	47.8
G3	152	14.0
*Infiltration level*		
In situ	72	6.7
Dermis	489	45.2
Hypodermis	294	27.2
Muscle	77	7.1
Bone/cartilage	49	4.5
Ulceration	580	53.6
Necrosis	5	0.5
Perineural invasion	80	7.4
Angiolymphatic invasion	9	0.8
Desmoplastic growth	0	0

Data are expressed as absolute and relative values

**Table 2:** Report of missing data in the 1082 cSCC tumors

Type	Variable	n=missing	n=valid
*Patient*	Gender	0	1082
	Age	0	1082
*Tumor*	Margin status	8	1074
	Tumor location	2	1080
	Differentiation	34	1048
	Infiltration level	101	981
	Desmoplastic growth	0	1082
	Perineural invasion	1	1081
	Necrosis	0	1082
	Ulceration	0	1082
	Angiolymphatic invasion	0	1082
	Site of incomplete margin	3	1079^a^
	Site closest margin	66	1016
	Category closest margin [mm]	72	1010
	Category furthest margin [mm]	634	448
	Tumor diameter	174	908
	Tumor thickness	161	921
*Procedural*	Incisional biopsy	0	1082
	Additional sample taken	0	1082
	Additional sample free	0	1082
	Clinical indication	407^b^	675

a The [sites of incomplete margins] that are complete are labeled as not applicable and are included in the total of 1079.

b When a diagnostic biopsy (n=83) was performed, the clinical indication was left blank and classified as missing in the above report.

Significant associations for incomplete margin status and tumor variables were observed for site subgroups ear (p=0.006) and peri-orbital zone (p=0.029); differentiation grade G3 (p=0.005); infiltration level beyond the dermis: hypodermis (p<0.001), muscle (p=0.013), and bone/cartilage (p<0.001); presence of perineural invasion (p=0.041); angiolymphatic invasion (p=0.046); and ulceration (p=0.010). Procedural variables showed a significant association between incomplete margin status and an additional sample excision (p<0.001) and additional sample not free (p<0.001). Diagnostic biopsy taken demonstrated a statistically significant association with complete excision (p=0.041). The Mann–Whitney U test was conducted and demonstrated a significant difference between incomplete margins and tumor diameter (U=45712.0, p≤0.001) and tumor thickness (U=54044.5, p<0.001). No association was observed for the patient variables gender and age (Table 3).

**Table 3:** Patient-, tumor-, and procedural variables for complete and incomplete excision.

Variable	Complete (946)	Incomplete (128)	Chi^2^	df	P-value^a^
	*n/complete (%)*	*n/incomplete (%)*			
Gender	M: 721/946 (76.2)	M: 98/ 128 (76.6)	0.007	1	0.931
	F: 225/946 (23.8)	F: 30/128 (23.4)			
Diagnostic biopsy taken	Yes: 78/946 (8.2)	Yes: 4/128(3.1)	4.191	1	**0.041**
	No: 868/946 (91.8)	No: 124/128 (96.9)			
Clinical indications			0.505	4	0.973
	SCC: 290/587 (49.4)	SCC: 40/81 (49.4)	0.019	1	0.891
	BCC: 152/587 (25.9)	BCC: 19/81 (23.5)	0.126	1	0.722
	Not malignant: 19/587 (3.2)	Not malignant: 3/81(3.7)	0.063^b^	1	0.802
	Epithelioma (not well defined): 125/587 (21.3)	Epithelioma (not well defined): 19/81 (23.4)	0.258	1	0.611
	SCC/Merkel: 1/587 (0.2)	SCC/Merkel: 0/81 (0)	0.135^c^	1	0.713
Additional sample taken	Yes: 286/946 (30.2)	Yes: 63/128 (49.2)	18.528	1	**<0.001**
	No: 660/946 (69.8)	No: 65/128 (50.8)			
Additional sample free	Yes: 268/286 (93.7)	Yes: 39/64 (60.9)	54.319	1	**<0.001**
	No: 17/286 (5.9)	No: 25/64 (39.1)			
Tumor location groups			1.016^d^	2	0.602
	H&N: 812/945 (85.9)	H&N: 108/127 (85.0)	0.196	1	0.658
	Trunk: 26/945 (2.8)	Trunk: 2/127 (1.6)	0.624^b^	1	0.429
	Limbs: 107/945 (11.3)	Limbs: 17/127 (13.4)	0.429	1	0.513
Tumor location subgroups			24.546^e^	10	**0.006**
	Cheek: 90/945 (9.5)	Cheek: 8/127 (6.3)	1.448	1	0.229
	Ear: 157/945 (16.6)	Ear: 34/127 (26.8)	7.659	1	**0.006**
	Forehead: 70/945 (7.4)	Forehead: 5/127 (3.9)	2.118	1	0.146
	Limbs: 107/945 (11.3)	Limbs: 17/127 (13.4)	0.429	1	0.513
	Neck: 18/945 (1.9)	Neck: 3/127 (2.4)	0.114^b^	1	0.735
	Nose: 48/945 (5.1)	Nose: 10/127 (7.9)	1.655	1	0.198
	Peri-oral: 48/945 (5.1)	Peri-oral: 4/127 (3.1)	0.930	1	0.335
	Peri-orbital: 35/945 (3.7)	Peri-orbital: 10/127 (7.9)	4.750	1	**0.029**
	Scalp: 270/945 (28.6)	Scalp: 21/127 (16.5)	8.405	1	**0.004**
	Temple: 76/945 (8.0)	Temple: 13/127 (10.2)	0.668	1	0.414
	Trunk: 26/945 (2.8)	Trunk: 2/127 (1.6)	0.624^b^	1	0.429
Infiltration level			63.243	4	**<0.001**
	In situ: 67/854 (7.8)	In situ: 5/120 (4.2)	1.819	1	0.177
	Dermis: 457/854 (53.5)	Dermis: 27/120 (22.5)	33.730	1	**<0.001**
	Hypodermis: 239/854 (28.0)	Hypodermis: 55/120 (45.8)	17.776	1	**<0.001**
	Muscle: 61/854 (7.1)	Muscle: 16/120 (13.3)	6.204	1	**0.013**
	Bone/cartilage: 30/854 (3.5)	Bone/cartilage: 17/120 (14.2)	27.538	1	**<0.001**
Differentiation grade			15.044	3	**0.002**
	In situ: 84/916 (9.2)	In situ: 5/124 (4.0)	3.669	1	0.055
	G1: 264/916 (28.8)	G1: 23/124 (18.5)	5.687	1	**0.017**
	G2: 447/916 (48.8)	G2: 68/124 (54.8)	1.558	1	0.212
	G3: 121/916 (13.2)	G3: 28/124 (22.6)	7.787	1	**0.005**
Ulceration	Yes: 492/946 (52.0)	Yes: 82/128 (64.1)	6.584	1	**0.010**
	No: 454/946 (48.0)	No: 46/128 (35.9)			
Perineural invasion	Yes: 64/946 (6.8)	Yes: 15/127 (11.8)	4.179	1	**0.041**
	No: 882/946 (93.2)	No: 112/127 (88.2)			
Angiolymphatic invasion	Yes: 6/946 (0.6)	Yes: 3/128 (2.3)	3.965^b^	1	**0.046**
	No: 940/946 (99.4)	No: 125/128 (97.7)			
Necrosis	Yes: 3/946 (0.3)	Yes: 2/128 (1.6)	3.774^c^	1	0.052
	No: 943/946(99.7)	No: 126/128 (98.4FIGURE)			
Desmoplastic growth	Yes: 0/946 (0)	Yes: 0/128 (0)	NA	NA	*constant*
	No: 946/946 (100)	No: 128/128 (100)			
Age	Mean rank 534.83	Mean rank 557.20	NA	NA	0.444
	Median 81.0 [range 36-99]	Median 80.5 [range 45-97]			
Macro-scopic dimension (cm)	Mean rank 440.50	Mean rank 534.37	NA	NA	**<0.001**
	Median 2 cm [range 0.20-12.00 cm]	Median 2.6 cm [range 0.50-9.00 cm]			
Tumor thickness (mm)	Mean rank 447.27	Mean rank 590.95	NA	NA	**<0.001**
	Median 4 mm [range 0.00-30.00 mm]	Median 6 mm [range 0.00-25.00 mm]			

Chi^2^= Pearson chi-squared test, M, male: F, female.

Categorical data are expressed in absolute and relative values. Continuous data are expressed in mean rank and median [range].

The significance level alpha is 0.050.

a All p-values for categorical variables were calculated using the Pearson chi-squared test for and p-values for continuous variables were tested using the Mann–Whitney U Test.

b 1 cells (25.0%) have expected count less than 5.

C 2 cells (50%) have expected count less than 5.

d 1 cells (16.7%) have expected count less than 5

e 2 cells (9.1%) have expected count less than 5.

Univariable logistic regression was conducted to assess the associations of independent factors with incomplete margin status. The set included all variables analyzed, and odds ratio (OR) are provided. No prior diagnostic biopsy (OR 2.786, p=0.049), additional sample taken (OR 0.447, p<0.001), additional sample free (OR 10.143, p<0.001), tumor location subgrouped (p=0.010), tumor diameter (OR 1.267, p<0.001), tumor thickness (OR 1.130, p<0.001), infiltration level (p<0.001), differentiation grade (p=0.003), ulceration (OR 0.608, p=0.011), and perineural invasion (OR 0.542, p=0.044) were found to be independent significant predictors for incomplete excision (Table 4).

**Table 4:** Univariable logistic regression with predictor variables tested one by one for incomplete excision

Predictor variable [reference group]	p-value	OR	95% CI
*Gender [M]*	0.931	0.981	0.635-1.516
*Age*	0.281	1.011	0.991-1.033
*Diagnostic biopsy taken [yes]*	**0.049**	2.786	1.002-7.743
*Clinical indication*^b^*[BCC]*	0.947	NA^a^	NA^a^
SCC	0.740	1.103	0.618-1.971
Not malignant	0.726	1.263	0.342-4.671
Epithelioma (not well defined)	0.572	1.216	0.617-2.397
*Additional sample taken [yes]*	**<0.001**	0.447	0.308-0.649
*Additional sample free [yes]*	**<0.001**	10.143	5.028-20.461
*Tumor location groups [trunk]*	0.607	NA^a^	NA^a^
Head and neck	0.460	1.729	0.405-7.387
*Limbs*	0.352	2.065	0.449-9.506
*Tumor location subgroups [trunk]*	**0.010**	NA^a^	NA^a^
Cheek	0.860	1.156	0.231-5.780
Ear	0.172	2.815	0.638-12.432
Forehead	0.932	0.929	0.170-5.085
Limbs	0.352	2.065	0.449-9.506
Neck	0.422	2.167	0.328-14.305
Nose	0.220	2.708	0.552-13.300
Peri-oral	0.929	1.083	0.186-6.317
Peri-orbital	0.108	3.714	0.749-18.411
Scalp	0.989	1.011	0.224-4.555
Temple	0.313	2.224	0.470-10.517
*Tumor diameter (cm)*	**<0.001**	1.267	1.137-1.412
*Tumor thickness (mm)*	**<0.001**	1.130	1.084-1.179
*Infiltration level [muscle]*	**<0.001**	NA^a^	NA^a^
*In situ*	0.020	0.285	0.098-0.823
*Dermis*	<0.001	0.225	0.115-0.442
*Hypodermis*	0.681	0.877	0.470-1.637
*Bone/ cartilage*	0.063	2.160	0.960-4.859
*Differentiation grade [in situ]*	**0.003**	NA^a^	NA^a^
*G1*	0.454	1.464	0.540-3.970
*G2*	0.050	2.556	1.001-6.527
*G3*	0.007	3.888	1.442-10.478
*Ulceration [yes]*	**0.011**	0.608	0.415-0.892
*Perineural invasion [yes]*	**0.044**	0.542	0.299-0.983
*Angiolymphatic invasion [yes]*	0.063	0.266	0.066-1.077
*Necrosis [yes]*	0.080	0.200	0.033-1.211

OR, odds ratio, M, male: n.a., not applicable

The significance level alpha is 0.050

a not applicable as the variable has more than two groups, specified OR for each category within statistically significant (p<0.05) independent variables are provided.

b The indication category [SCC/Merkel] n=1 is excluded from the analysis.

## Discussion

Cutaneous squamous cell cancer represents 25% of all keratinocyte carcinomas and its incidence is rising.^2^^,^^5^, ^6^, ^7^ Owing to the increasing life expectancy, the time required for ultraviolet radiation (UVR) damage to evolve into skin cancer has increased.^15^ Surgical excision with histological examinations of the margins is the first-choice of treatment for cSCC. Guidelines lack a consensus on the surgical margins recommended.^16^ European guidelines recommend 5 mm and 6-10 mm for peripheral surgical margins in low- and high-risk tumors, respectively, the deep margin should include the subcutis.^8^ The American NCNN guidelines recommend a 4-6 mm surgical margin in low-risk cSCC and wider margins for high-risk cSCC without specified recommendations on these; for deep margins, they recommend a depth of the mid-subcutis. Furthermore, they strongly recommend Mohs micrographic surgery (MMS) in high-risk tumors as margin status is examined upon excision.^17^ However, this option is not available throughout all medical centers and high-risk cSCCs are therefore frequently treated using WLE. To minimize the risk of local recurrence, deep subclinical progression and metastasis, it is important to obtain a radical surgical excision with clinical and microscopic control of excision margins.^8^^,^^17^ An incomplete excision is defined as the presence of cancer cells at the surgical margin; few studies expand this definition to residual cancer cells present within 1 mm of the surgical margin.^11^ To this date, only few studies have analyzed risk factors associated with incomplete excision of cSCC in a large cohort. In addition, systematic reviews showed high heterogeneity in study design resulting in a lack of conclusiveness.

In this cohort with 1082 cSCCs in 837 patients, tumors were the most frequently located in the H&N area (85.7%) and had differentiation grades G2 or G3 (61.8%). These findings are easily explained by the fact that, according to the Hospital's Diagnostic-Therapeutic Pathway, lesions located in areas of high cosmetic value and large or aggressive lesions that may require complex reconstruction are referred to the Plastic Surgery Department by dermatologists or GPs. Recent studies investigating the risk factors associated with incomplete excision of keratinocyte carcinomas in larger cohorts have been published; however, in contrast with this study, these studies involve patients treated in a primary care setting, with less aggressive lesions and/or located in low-aesthetic-value body sites.^12^, ^13^, ^14^ For the aforementioned purposes, to the authors’ knowledge, this study presents the largest series, to date, of cSCCs requiring treatment by plastic surgery.

In this study, the incomplete excision rate for cSCC was 11.8%. This close to the recent pooled incomplete excision rate of 13% found in the literature.^11^ Several studies found a positive association between the rate of incomplete excisions and worse differentiation grade, larger dimensions, higher tumor thickness, and tumor located in the H&N area.^11^ The present study confirmed the frequently associated risk factors for incomplete excision of cSCCs such as differentiation grade, infiltration depth, and level and tumor diameter. Several additional risk factors have been found. The analysis of all risk factors found will be discussed in the sections below.

### Patient risk factors

This study did not demonstrate a significant association between incomplete excisions and patient-related variables, such as age and gender. This association is rarely found in literature.^11^ The gender distribution within this study is unequal with a prevalence male patients (76.2%). This finding is in line with a prior study within our center in which this topic is addressed.^18^

### Preoperative risk factors

These risk factors can be considered prior to surgery by performing physical examination alone. For location, contrary to the findings of most literature, we did not find an association between tumors in the H&N area and incomplete excisions.^11^^,^^12^^,^^14^ In this series, the percentage of H&N tumors was 85.7%, representing a select population, with only 14.2% having tumors outside of this area. This lack of variation in tumor locations could explain why the study did not show the H&N area to be significantly associated with incomplete excisions. However, while analyzing the subdivisions, location at the ear and peri-orbital zones were found to be significantly associated with incomplete excisions. In this regard, ear localization was found to be associated with incomplete excisions earlier.^14^^,^^19^ The scalp location was significantly associated with higher rate of complete excisions. In the authors’ opinion, the scalp defects can be addressed via a wide range of reconstructive choices. First, the vascular anatomy of the scalp allows flap-based reconstruction of larger defects. Additionally, when flaps are contra-indicated or offer insufficient coverage, a Dermal Regeneration Template can be used, with good cosmetic results providing more anatomical flexibility in reaching adequate surgical margins adhering to the AIOM guidelines.^20^

In this study, a significant association was found for tumor diameter (Figure 6), this is consistent with the scientific literature. In global guidelines, the diameter, among other tumor characteristics, classifies tumors into low risk (>2 cm) and high risk (>2 to <4 cm) and in some guidelines very high risk (>4 cm) lesions.^8^^,^^17^ High- or very high-risk tumors are expected to have clinically and biologically more aggressive behavior; therefore, macroscopically larger tumors require wider surgical margins.^8^^,^^17^ Lastly, an association between the presence of ulceration and incomplete excisions was found. To the author's knowledge, this characteristic is not mentioned in studies on risk factors for margin status in cSCC in recent literature.

### Procedural risk factors

These factors were studied to establish a potential relationship between the surgical procedure based upon the suspected clinical diagnosis and results of the pathological analysis. Although no relation between incomplete excisions and clinical indication was found, a significant association emerged between incomplete excisions and the fact that additional sample(s) were taken during the same surgical procedure after the main excision. As in the setting of this study, the opportunity for frozen section pathological analysis is not a routine practice, with additional specimen being excised t substantial frequencies (32.6%). In this study, the excision was labeled as “incomplete” whenever the lesion was present at any of the surgical margins of the main specimen, regardless of the additional sample(s) status. A significant association between incomplete excision and presence of tumor cells (classified: not free) in additional tissues does suggest a link between the clinician's doubts during surgery and the pathological outcome. Further analysis is needed to draw precise conclusions on this topic. As the origin of these additional tissues varies widely, establishing a relation between these and incomplete excisions is challenging as it requires context.

The diagnostic biopsy rate in this study was 7.7%. European guidelines recommend performing diagnostic biopsies in all clinically suspicious lesions, as this facilitates prognostic classification and correct management of cSCCs.^21^ The study corroborates this, as a prior diagnostic biopsy was found to be a significant risk factor for complete excision and thus preventive for incomplete excision. The presumption that a surgeon could feel more confident and entitled in expanding the surgical margins in case of a positive cSCC diagnosis provides a possible explanation. Accordingly, the authors addressed the importance of a biopsy-confirmed diagnosis, especially in case of lesions of large dimensions and/or those at high-risk sites.^8^ This study, a with a low diagnostic rate of 7.7%, illustrates that by performing a biopsy, the risk of incomplete excision can be decreased and sheds light on the importance of this step in the diagnostic process.

### Pathological risk factors

Infiltration level beyond the dermis and larger tumor thickness were found to be significant risk factors for incomplete margin status (Figure 7). G3 differentiation grade was found to be a significant risk factor for incomplete excision whereas differentiation grade G1 appeared to be a significant predictor for complete excision. This association was not found in other recent studies.^11^ In this study, grading was moderate to poor in more than 50% of the cases and infiltration beyond the dermis was reported in 38.8% of the cases. When comparing these figures to other large-cohort studies, histopathological tumor cohort population appeared to represent a specified, higher risk, patient population.^12^, ^13^, ^14^ This circumstance could explain the discrepancy in risk factor differentiation grade in this study compared to other large cohorts. Lastly, perineural- and angiolymphatic invasion were found to be significantly associated with incomplete excisions; for angiolymphatic invasion, this association was not confirmed in the logistic regression as the cell counts were low. To the authors knowledge, this is a new-found association as perineural invasion was included in this risk factor analysis only once, where it showed no association, and angiolymphatic invasion was not mentioned in prior studies for incomplete excisions of cSCC.^11^^,^^12^ Lastly, by not performing frozen section pathological analysis or MMS within our center, the findings of our study are especially relevant to similar centers. Further research on incomplete excision using these techniques is needed to relate the findings of our study to these methods.

### Influence of COVID

With the Bergamo province being the center of the Italian COVID-19 outbreak, the Papa Giovanni XXIII Hospital was severely affected and most surgeons put down the scalpel to help at the COVID-19 warfront.^22^ The consequences of the pandemic have been previously researched and a significant increase was found in the dimensions of keratinocyte carcinomas excised within our center.^23^ As large tumor diameters are considered a risk factor for incomplete excisions, the assumption that the incomplete excision rate would have increased post-COVID was tested. This assumption was disproved as incomplete excision rates declined in 2020 and 2021 (Figure 5). In the authors’ opinion, the absolute need for optimization of operating rooms, equipment, and staff might have led surgeons to use wider surgical margins to minimize the risk of further re-excision surgeries.

## Limitations and future perspectives

Some limitations of this study are identified. First, the authors highlight the unbalanced distribution of tumor locations within the study population. Despite highlighting the importance of big data while constituting the study design, the focus on H&N tumors and lack of variation in the distribution of tumor sites, resulted in low cell counts for other body locations. In addition, the number of risk factors found to be significant exceeded the potential variable number for a multivariable logistic regression, which could have provided a more detailed risk analysis.

The authors acknowledge a gap in this analysis, represented by the unexplored role of Mohs surgery in reducing the amount of incomplete excisions. MMS is a renowned paramount asset in the toolbox of the plastic surgeon. However, the intraoperative study of surgical margins comes with higher costs and longer surgical times. In centers where the surgical waiting lists for skin cancer treatment are overcrowded, the need for optimization of OR times and resources may turn MMS an occasional—rather than routine—practice. In this scenario, this study aimed to optimize the chances of WLE to be as effective as possible, by improving radicality. Considering risk factors is important, but it cannot replace the verification of excision margins and depth of excision using frozen sections or MMS.

The main limitation of this study is the design: this is a single-center retrospective study and is therefore limited in terms of the conclusions that can be drawn on an international scale. With the first pilot retrospective study phase completed; it is in the authors’ interest to extend this analysis to a multi-center setting. Several topics, such as the impact of additional samples on surgical radicality; the definition, risk factors and management of close margins; the surgical outcomes in terms of disease-free survival and 5-year local recurrence rates, remain unresolved and will be the focus for future research. To decrease risks for local recurrence, deep subclinical progression, and metastasis, risk factors should be considered prior to primary surgery. With this retrospective study, the authors identified few significant preoperative and clinically detectable factors (i.e., tumor location and diameter, ulceration) significantly associated with a higher rate of incomplete excision. In addition, as a biopsy-confirmed diagnosis was significantly associated with clear margins at excision, relying on more diagnostic biopsies should be encouraged, especially in high-risk lesions.

## Conclusion

Clinically and statistically significant risk factors for incomplete excision of cSCC include absence of prior diagnostic biopsy, location on the ear and peri-orbital zone, differentiation grade G3, infiltration level beyond the dermis, tumor diameter, tumor thickness, perineural invasion, and ulceration. Risk factors, focusing on preoperatively and clinically detectable risk factors, should be considered in the management of cutaneous squamous cell carcinoma treatment to decrease the risk of incomplete excision and thereby minimize the risk of local- or distant recurrence and metastasis.

## Other information

The study has no funding. The authors have no conflicts of interest to declare.

## Appendix Supplementary materials

Image, application 1
