# Supplementary material for: Risk factors for incomplete excision of cutaneous squamous cell carcinoma: A single-center study performed on 1082 excisions in Northern Italy
Source: JPRAS Open. 2025 Mar 6;44:316–30. doi: 10.1016/j.jpra.2025.02.007 (PMC12018306; doi:10.1016/j.jpra.2025.02.007)

**Supplements**

Supplementary Table 1: Clinicopathological data collected from histopathology reports

| **Category** | **Data recorded** | **Recording options** |
| --- | --- | --- |
| *Patient* | gender  age^a^ | [female/ male]  [years] |
| *Tumour* | Margin status  site of incomplete margin  site of closest margin  distance of closest margin  ulceration  necrosis  desmoplastic growth  perineural invasion  angiolymphatic invasion  tumour location groups  tumour location subgroups  differentiation grade  infiltration level  tumour diameter  infiltration depth | [complete/ incomplete]  [lateral/ peripheral/ both]  [lateral, deep]  [0, >0-1, >1-4, >4-6, >6-8, >8] [mm]  [yes/no]  [yes/no]  [yes/no]  [yes/no]  [yes/no]  [head & neck, limbs, trunk]  [scalp, ear, limbs, temple, cheek, forehead, periorbital, peri-oral, nose, trunk, neck]  [in situ, G1=well, G2=moderate, G3=poor]  [in situ, dermis, hypodermis, muscle, bone/ cartilage]  Diameter [cm]  Thickness [mm] |
| *Procedural* | biopsy  clinical indication  additional tissue taken  additional tissue free  type of procedure | [yes/no] [SCC, BCC, not malignant, SCC/ Merkel, epithelioma (not-well defined)]  [yes/no]  [yes/no]  [outpatient clinic day treatment, general operating room] |

^a^age was calculated as age in years at the moment of primary surgery

Fig 1 Tumour sites subdivided into subgroups and groups


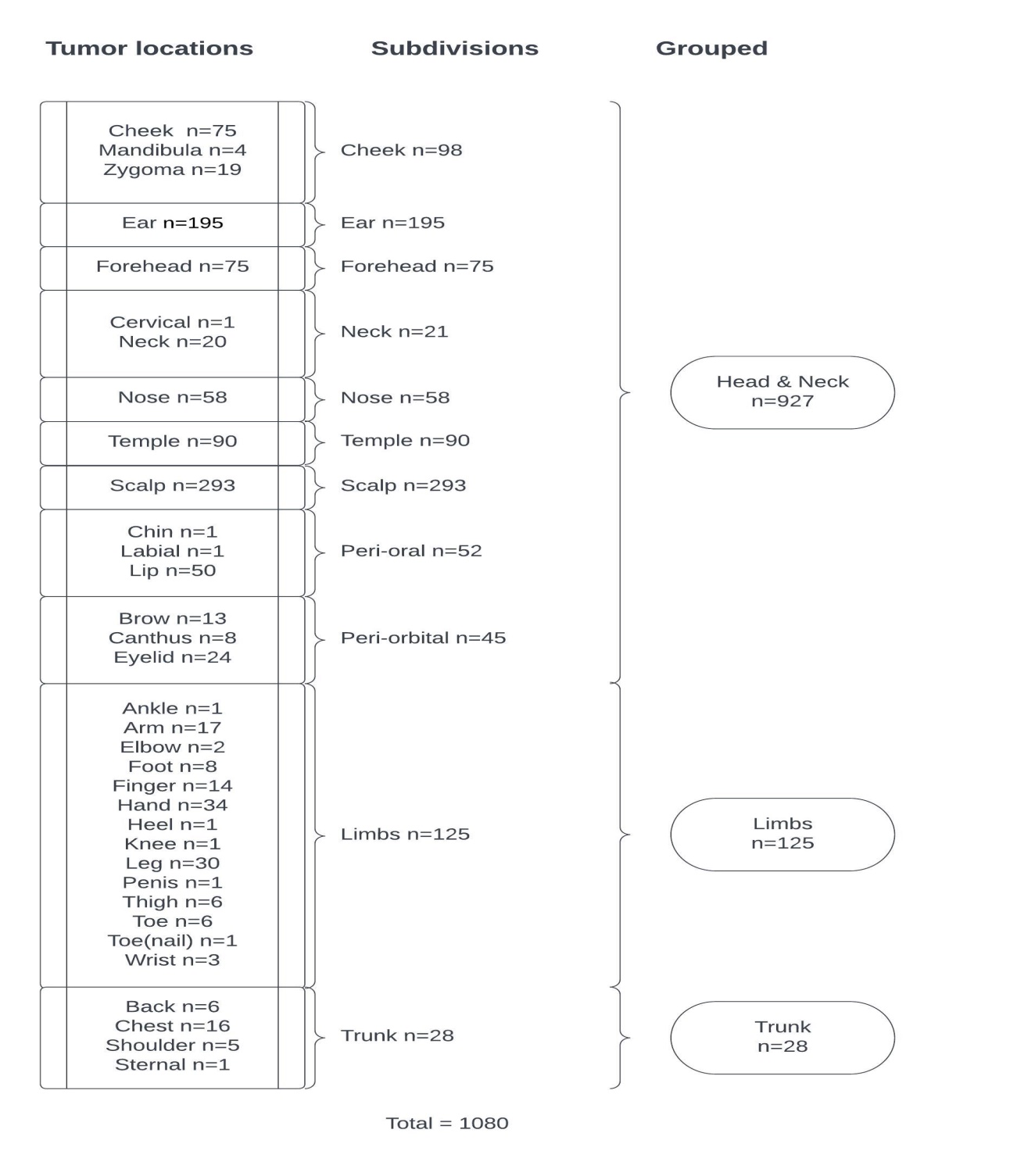


Fig 2 Facial subdivision for tumour sites


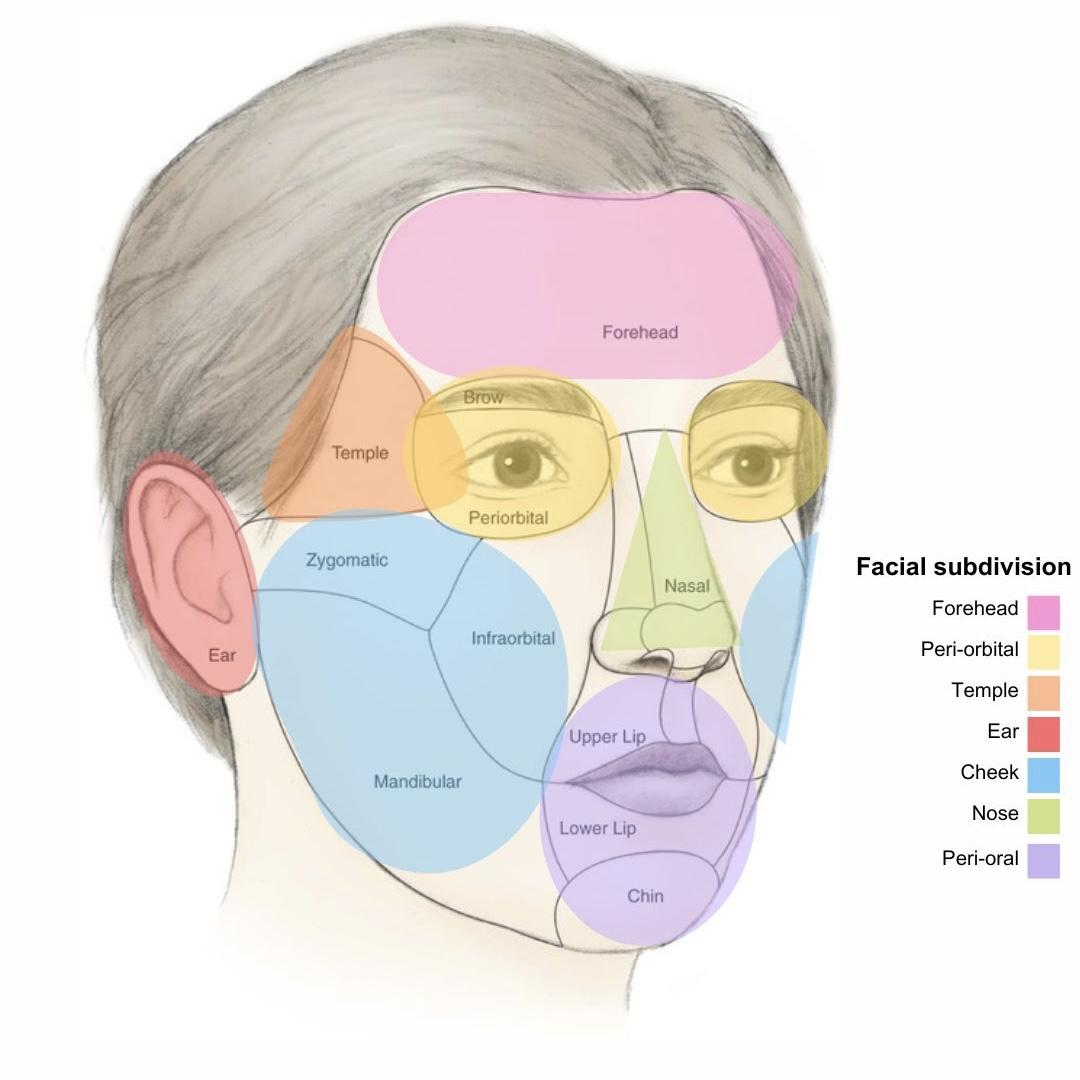

Supplement: Supplementary file 1 [file mmc1.docx]
